# Downregulation of Cypher induces apoptosis in cardiomyocytes via Akt/p38 MAPK signaling pathway

**DOI:** 10.7150/ijms.48872

**Published:** 2020-08-27

**Authors:** Tianming Xuan, Dongfei Wang, Jialan Lv, Zhicheng Pan, Juan Fang, Yin Xiang, Hongqiang Cheng, Xingxiang Wang, Xiaogang Guo

**Affiliations:** 1 Department of Cardiology, The First Affiliated Hospital, Zhejiang University School of Medicine, Hangzhou, China.; 2 Department of Pathology and Pathophysiology, Zhejiang University School of Medicine, Hangzhou, China.

**Keywords:** apoptosis, Cypher, dilated cardiomyopathy, Akt, p38 MAPK

## Abstract

**Background:** Dilated cardiomyopathy (DCM) is considered as the most common form of non-ischemic cardiomyopathy with a high mortality worldwide. Cytoskeleton protein Cypher plays an important role in maintaining cardiac function. Genetic studies in human and animal models revealed that Cypher is involved in the development of DCM. However, the underlying molecular mechanism is not fully understood. Accumulating evidences suggest that apoptosis in myocytes may contribute to DCM. Thus, the purpose of this study is to define whether lack of Cypher in cardiomyocytes can elevate apoptosis signaling and lead to DCM eventually.

**Methods and Results:** Cypher-siRNA sufficiently inhibited Cypher expression in cardiomyocytes. TUNEL-positive cardiomyocytes were increased in both Cypher knockdown neonatal rat cardiomyocytes and Cypher knockout mice hearts, which were rare in the control group. Flow cytometry further confirmed that downregulation of Cypher significantly increased myocytes apoptosis *in vitro*. Cell counting kit-8 assay revealed that Cypher knockdown in H9c2 cells significantly reduced cell viability. Cypher knockdown was found to increase cleaved caspase-3 expression and suppress p21, ratio of bcl-2 to Bax. Cypher-deficiency induced apoptosis was linked to downregulation of Akt activation and elevated p-p38 MAPK accumulation. Pharmacological activation of Akt with SC79 attenuated apoptosis with enhanced phosphorylation of Akt and reduced p-p38 MAPK and Bax expression.

**Conclusions:** Downregulation of Cypher participates in the promotion of cardiomyocytes apoptosis through inhibiting Akt dependent pathway and enhancing p38 MAPK phosphorylation. These findings may provide a new potential therapeutic strategy for the treatment of DCM.

## Introduction

Cypher/ZASP, encoded by LIM domain binding 3 (*LDB3*), is a cytoskeletal PDZ-LIM protein predominantly expressed in cardiac and skeletal muscle 1. It is localized at the Z-line by directly complexing with α-actinin-2 through its PDZ domain to maintain sarcomere integrity during muscle contraction 2. And the LIM domains are involved in the binding to a network of proteins such as protein kinase acting as a signaling center 3. Genetic studies have revealed that mutations in the* LDB3* gene are shown to be associated with human myopathies such as skeletal myopathy, isolated non-compaction of the left ventricular myocardium, hypertrophic cardiomyopathy and dilated cardiomyopathy (DCM) 4. Moreover, the *LDB3* gene, mapped on chromosome 10q22.3-10q23.2, overlaps with a DCM locus 5. DCM, the most common cause of congestive heart failure, is a cardiac muscle disease characterized by left ventricular dilation with systolic dysfunction which often necessitates cardiac transplantation 6. Although the pathogenesis of DCM are highly heterogeneous, up to 35% of cases are caused by mutations, mainly in cytoskeletal and sarcomeric genes 7. Among them, Cypher/ZASP plays a critical role in the pathogenesis of DCM in both human and animal models 8-11.

In zebrafish, knockdown of Cypher results in a DCM phenotype which is characterized by cardiac dilation alongside with significant thinning of the ventricular wall 9. In mice, both global and cardiac-restricted Cypher knockout induce a severe form of DCM with heart failure, which lead to premature lethality 10. A similar phenotype is observed in a transgenic mouse model (S196L) 11. Moreover, Cypher deficiency induced DCM in mouse models leads to abnormalities of multiple signaling pathways. For example, calcineurin signaling is overactivated which is a Ser/Thr phosphatase of the L-type calcium channel (LTCC) in Cypher deficient mouse 12,13. Cypher also can tether protein kinase A (PKA) to the LTCC and phosphorylate Ser1928 through specifically interacting with PKA, termed an AKAP 13. The mechanosensing protein Ankrd2 and the nuclear phosphoprotein p53 are another two binding partners of Cypher 14. And they also found that the Cypher acts as a negative regulator to decrease the p53-mediated activation of Bax and MDM2 promoters 14. The DCM related D626N mutation of Cypher/ZASP increases its affinity to protein kinase C (PKC), further suggesting that Cypher/ZASP plays an important signaling role to maintain cardiac function 15.

Accumulating studies using zebrafish, mouse, and human genetics have revealed a pivotal role of Cypher/ZASP for maintaining cardiac structure and function. However, the molecular mechanisms underlying the deficiencies in Cypher/ZASP resulting in DCM are still poorly understood. Further investigations are essential for providing initial insights into the pathophysiology for Cypher/ZASP related DCM as well as developing new therapeutic strategies to prevent progression and mortality due to DCM. Therefore, we investigated whether DCM induced by cypher-deficiency is mediated by apoptosis which plays an important role in the pathogenesis of DCM.

## Materials and Methods

### Experimental animals

All procedures conformed to the Guide for the Care and Use of Laboratory Animals published by the US National Institutes of Health (NIH Publication no. 85-23, revised 1996) and were approved by the ethics committee of the First Affiliated Hospital, School of Medicine, Zhejiang University. C57BL/6J global Cypher knockout mice were a generous gift of Dr. Cheng. All mice were housed and bred in an accredited facility, which was maintained at 20-25°C, 55% humidity, with a 12/12 hrs light/dark cycle. Mice were sacrificed at birth and the hearts were collected.

### Neonatal rat cardiomyocytes (NRVCM) culture

Neonatal rat cardiomyocytes were prepared from 1-3 days old Sprague-Dawley rats bred at Zhejiang Academy of Medical Sciences by adapting previously published protocols 16.

### Cell culture

HEK293T cell line and H9c2 cell line were purchased from Chinese Academy of Sciences. Cells were cultured in high glucose DMEM supplemented with 10% FBS, 100 U/mL of penicillin/streptomycin. Cells were incubated at 37°C in the presence of 5% CO_2_. For some groups, the H9c2 cells were treated with Akt agonists SC79 (HY-18749, 10 mM, 30 mins, MCE), and then the cells were harvested in 30 mins for Western blot analysis.

### siRNA transfection

siRNA transfections were performed with Lipofectamine RNAiMAX reagent (Invitrogen, USA) following the manufacturer's instructions. The siRNA to lipofectamine RNAiMAX reagent concentration used was 10 pmol to 7.5 µL. After incubation for 48 hrs, the H9c2 cells or primary neonatal rat cardiomyocytes were used for subsequent experiments. The siRNA targeting rat Cypher (5'-GGAACAGCCUCUUCCACAUTT-3') or nontargeting scrambled siRNA (5'-UUCUCCGAACGUGUCACGUTT-3') were synthesized by GenePharma (Shanghai, China).

### TdT-mediated dUTP nick end labeling (TUNEL) assay

TUNEL assay was performed using the *In situ* Cell Death Detection Kit (Roche Diagnostics, Mannheim, Germany) to detect apoptotic cells in the mouse hearts (experiments were accomplished by Wuhan Seville Biological Technology, Ltd.). Positive staining which exhibited dark brown, brownish and light yellow nuclei under a light microscope was analyzed by Quant center software. The cell apoptosis positive rate = number of apoptotic cells/number of total cells×100%. Apoptosis of neonatal rat cardiomyocytes was detected using the TUNEL FITC Apoptosis Detection kit (Beyotime) according to the manufacturer's instructions. In brief, cells were washed with PBS once and fixed in 4% paraformaldehyde for 30 mins. Cells were then treated with 0.3% Triton-X for 5 min, rinsed with PBS, and subsequently incubated with a mixture of Biotin-dUTP and the TdT enzyme in a humidified atmosphere for 60 mins at 37°C. Finally, DAPI was used to dye the nucleus. Finally, TUNEL positive cells were observed under fluorescence microscope.

### Cell counting kit-8 (CCK-8) assay

Si-Cypher-transfected, si-scramble-transfected and untreated H9c2 cells were seeded into 96-well plates (1×10^5^ cells/mL) for the CCK-8 cell proliferation assay (lot: K10183133EF5E, Beyotime). According to the manufacturer's instructions, cells were incubated with the CCK-8 reagent at 37°C for 2 hrs, with the absorbance of each sample scanned on a microplate reader equipped to read absorbance values at 450 nm.

### Assessment of apoptosis by flow cytometric analysis

Cells were treated with siRNA for 48 hrs. Following treatment, cells were stained with Annexin V/PI Apoptosis Detection Kit (catalogue: 556547, BD Biosciences, USA). Briefly, after digestion with 0.25% trypsin, cells were harvested by centrifugation, washed twice with cold PBS, and then resuspended in 500 μL 1×Binding Buffer at a concentration of 1×10^5^ cells/mL. The cells were then incubated with 5 µL Annexin V-FITC and 5 µL PI at room temperature in dark for 15 mins with gentle vortexing. Stained cells were analyzed by flow cytometry. Data analysis was performed with FlowJo 10.5.0 (FlowJo LLC). The experiment was repeated for three times.

### Quantitative Real time Polymerase Chain Reaction (RT-qPCR) analysis

Total RNA was extracted from cell samples using TRIzol reagent (Invitrogen; Thermo Fisher Scientific, Inc.). 500 ng of isolated RNA was reverse transcribed into cDNA using PrimeScript™ RT Master Mix (Takara Biotechnology Co., Ltd., China) according to the manufacturer's protocol. And RT-qPCR reaction systems were prepared using SYBR Green qPCR Master Mix (Vazyme Biotech Co. Ltd). The thermocycling conditions were: 95°C for 30 secs; followed by 39 cycles at 95°C for 10 secs and 60°C for 30 secs. Relative transcript abundance was normalized against GAPDH. The 2^-ΔΔCT^ method was used to calculate the relative transcript level.

### Western blotting

Western blotting was performed as previously described 17. Blots were incubated overnight at 4°C with following primary antibodies: GAPDH (Cell Signaling Technology, CST), LDB3 (Abcam), p21 (Abcam), Bax (Abcam), Bad (CST), phospho-Bad (Ser112, CST), Bcl-2 (Proteintech), caspase3 (CST), cleaved caspase-3 (CST), Akt (CST), phospho-Akt (Ser473, CST), phospho-Akt (Thr308, CST), p38 MAPK (CST), phospho-p38 MAPK (Thr180/Tyr182, CST), Erk1/2 (CST), phospho-Erk1/2 (Thr202/Tyr204, CST), SAPK/JNK (#9252, CST), phospho-SAPK/JNK (Thr183/Tyr185, CST), p53 (CST), GST (Abcam), HA (EΛRTH). The bands were quantified by image J software.

### Co-immunoprecipitation (Co-IP)

Plasmid expressing GST-Cypher and HA-Akt were co-transfected into HEK293T cells using Lipofectamine 2000 (Invitrogen, USA) according to the manufacturer's protocol. Cell lysates were collected 48 h post-transfection and subsequently incubated with GST-tag Cypher, HA-tag Akt, or control IgG antibodies overnight at 4°C on a rotator. Thereafter, protein A/G agarose beads were added for another 2 hrs at room temperature. The precipitates were then washed three times and boiled at 95°C for 10 mins. The eluted proteins were analyzed by western blot.

### Statistical analysis

The data were expressed as means ± standard deviations (SDs). Statistical comparisons between two groups were performed with an unpaired Student's *t* test. Statistical analysis was performed using GraphPad Prism 7.0 (GraphPad Software Inc., San Diego, CA, USA). *P* values < 0.05 were considered statistically significant.

## Results

### Knockdown of Cypher promoted cardiomyocyte apoptosis *in vivo* and vitro

**Figure 1:**
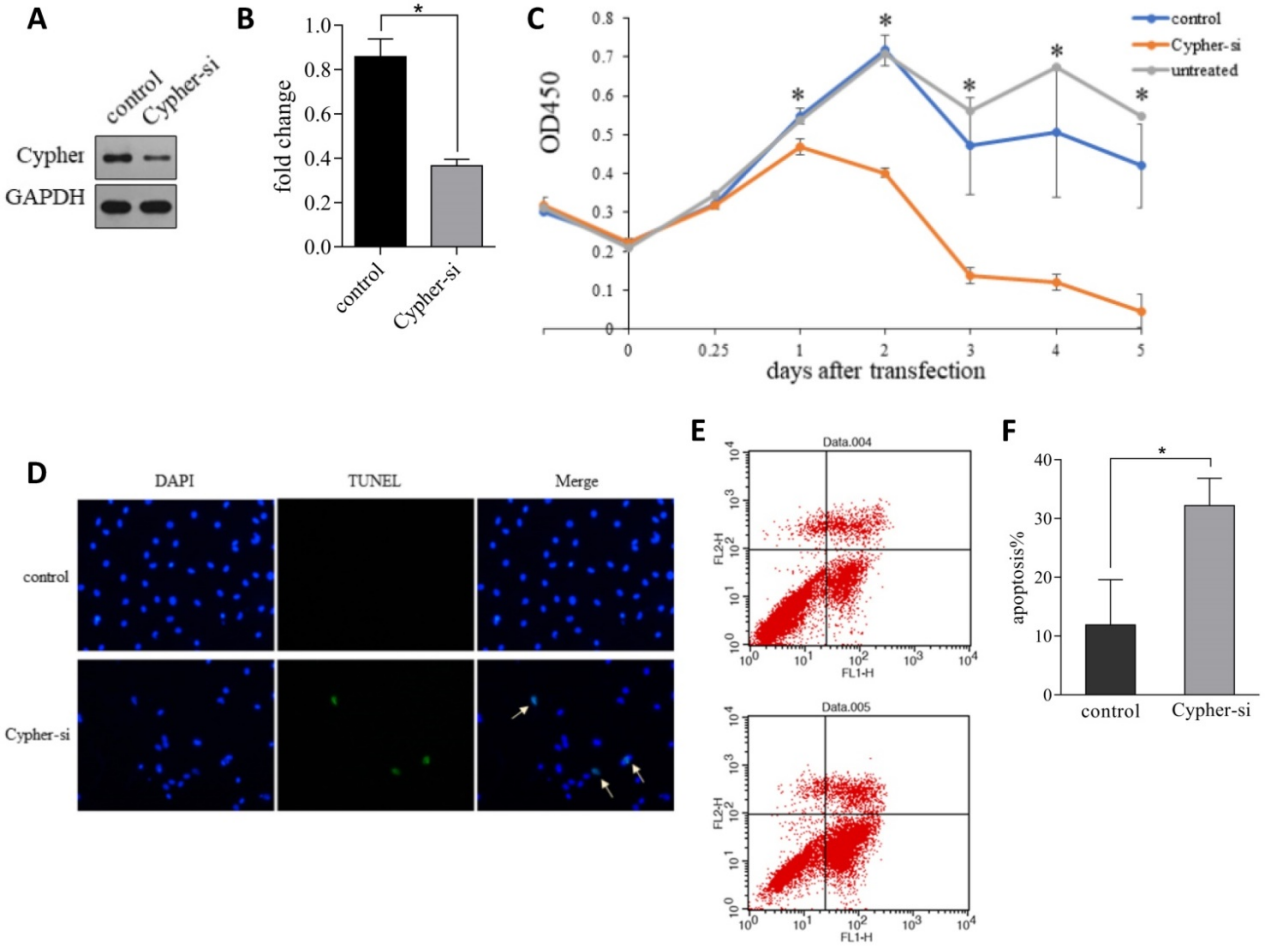
** Downregulation of Cypher induced apoptosis *in vitro*.** (**A and B**) Representative western blot bands and statistical results of Cypher protein expression normalized by GAPDH. *P < 0.05 versus control cells. (**C**) Cell viability was measured using the CCK-8 assay. *P < 0.05 Cypher-si versus control. (**D**) Representative images of TUNEL staining showing the apoptotic cells (stained in green) and nucleus (stained in blue with DAPI). Arrowheads in the pictures indicate apoptotic cell nuclei. (**E**) Annexin V-FITC/propidium iodide (PI) staining by flow cytometry was performed to detect the apoptosis of H9c2 cells. Early apoptosis (Annexin V-FITC+/PI), late apoptosis (Annexin V-FITC+/PI+), and necrosis (Annexin V-FITC/PI+). (**F**) The quantitative presentation of apoptotic cell population by Annexin V-FITC/PI staining. *P < 0.05 versus control. Experiments were repeated three times.

**Figure 2:**
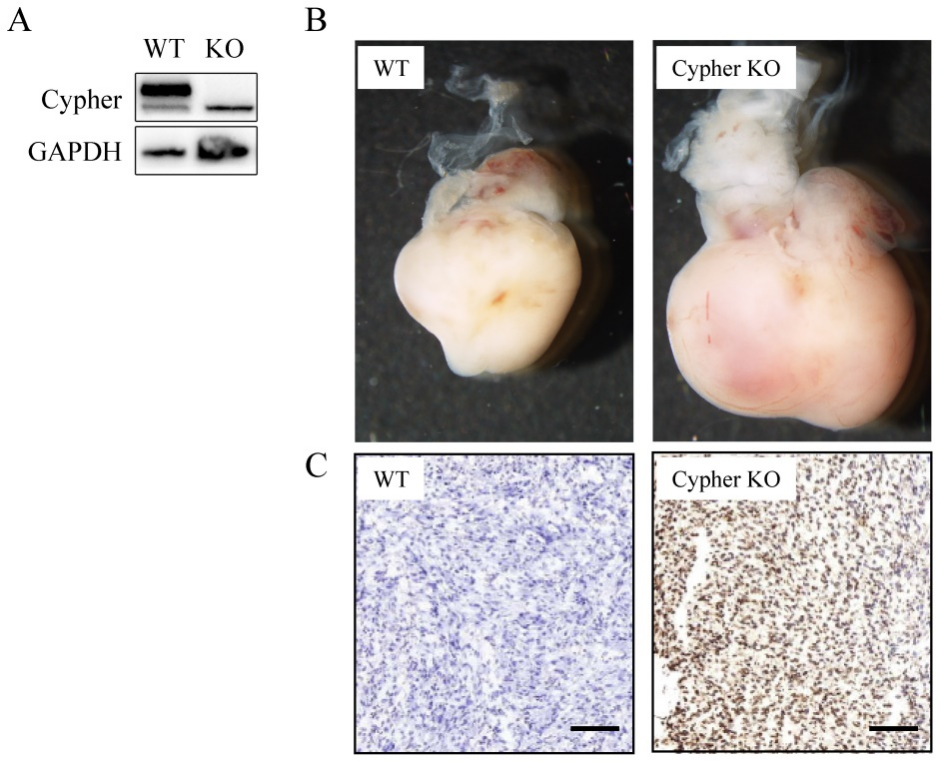
** Effects of Cypher in mice hearts.** (**A**) Representative western blot bands of Cypher protein expression was shown. (**B**) The effects of Cypher knockout on heart volume. (**C**) Representative TUNEL staining images from hearts of wild type mice (left) and Cypher knockout mice (right), scale bar: 100 µM.

To investigate the potential role of Cypher, the siRNA-mediated specific knockdown of Cypher was applied to downregulate its expression level. Scramble siRNA, a non-functional and non-targeting siRNA, was used as the control group. And the highly transfection efficiency was confirmed by western blotting. As shown in **Figure 1A and 1B**, the protein levels of Cypher were significantly diminished relative to the control group (*p*<0.05) and a decrease in intercellular adhesion and cell number, as well as cell shrinkage, were observed in cells treated with Cypher siRNA. Thus, CCK-8 assay was used to detect cell viability. Inhibition of Cypher in H9c2 cells efficiently reduced cell viability (**Fig. 1C**). The effect began to appear on the first day following Cypher knockdown, and continued to deteriorate until the end of the experiment which lasted for 5 days. To determine whether deficient Cypher was involved in NRVCMs apoptosis, the TUNEL staining and flow cytometry were performed. As shown in **Figure 1D**, knockdown of Cypher notably increased the proportion of TUNEL positive cells compared with the controls. Quantitative analysis using flow cytometry confirmed that knockdown of Cypher significantly increased the apoptotic rate of the primary cells. As shown in Fig.1E and 1F, the number of Annexin V+ and Annexin V+/PI+ cells, standing for early and late stage of apoptosis, were increased compared with the controls. To further assess the relationship between Cypher and cardiomyocyte, Cypher knockout mice were used (**Fig. 2A**). Morphologically, Cypher knockout mice exhibited larger heart than wild type littermates (**Fig. 2B**). As shown in **Figure 2C**, TUNEL-negative nuclei appeared blue, and TUNEL-positive nuclei were dark brown, brownish and light yellow. A marked increase in the number of TUNEL positive nuclei was observed in cardiac tissue of the Cypher knockout mice with apoptotic indices of 0.32, more than twice the wide type. Taken together, we strongly suggested that Cypher deficiency may induce cardiomyocytes apoptosis, which eventually causes dilated cardiomyocyte.

### Effect of Cypher-siRNA transfection on H9c2 cell apoptosis

**Figure 3:**
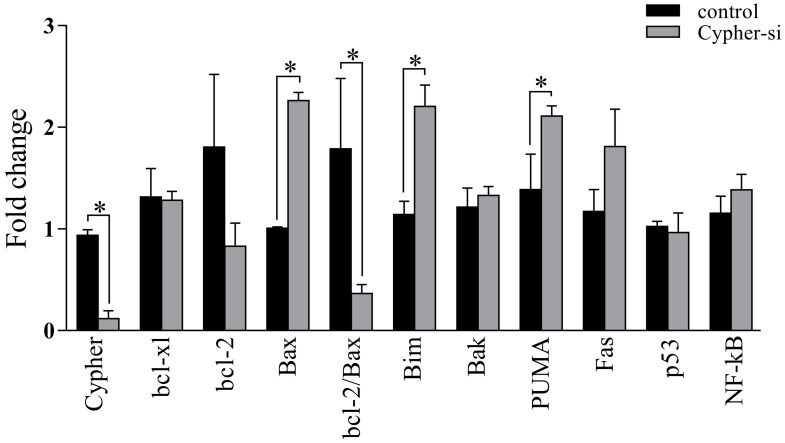
** Effects of Cypher knockdown on the mRNA expression of apoptosis-related proteins in H9c2 cells.** The mRNA levels of Cypher, bcl-xl, bcl-2, Bax, Bim, Bak, PUMA, Fas, p53, and NF-κB were detected by real-time polymerase chain reaction procedure. The relative levels of above mentioned mRNA were calculated by the values of ΔCt by normalizing with that of GAPDH. *P < 0.05 versus control. Experiments were repeated three times.

**Figure 4:**
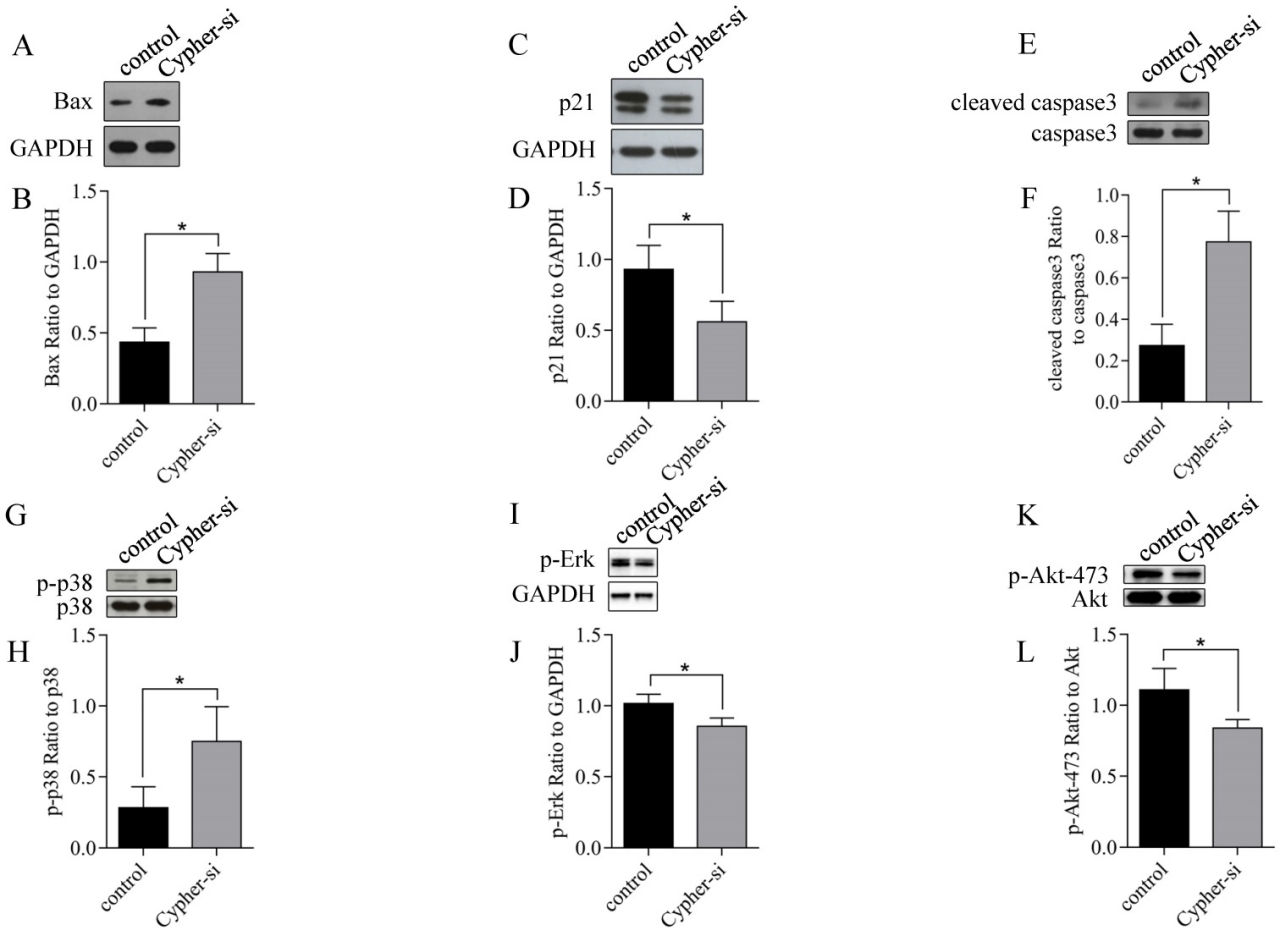
** Effects of Cypher knockdown on apoptosis regulatory protein expression in H9c2 cells.** (**A-L**) Representative western blot bands and statistical results of protein expression. *P < 0.05 versus control cells. Experiments were repeated three times.

As the knockdown of Cypher induced cell apoptosis, we employed RT-qPCR and western blot analysis to identify the expression of genes related to apoptosis following the reduction in Cypher expression. The results of RT-qPCR revealed that administration of Cypher siRNA resulted in decreased expression of bcl-2 and caused upregulation of apoptosis-inducing agents such as Bim, PUMA and Bax (**Fig. 3**). Then, the ratio of bcl-2 to Bax expression, which is the determining factor for the induction of apoptosis, was markedly decreased in Cypher siRNA treated H9c2 cells compared to controls. As expected, Western blotting displayed the similar results that the protein expression levels of Bax was upregulated in Cypher siRNA treated H9c2 cells (**Fig. 4A and 4B**). Furthermore, a significant decreased level of antiapoptotic protein p21 in Cypher siRNA treated H9c2 cells was detected (**Fig. 4C and 4D**). Increased apoptosis in Cypher knockdown H9c2 cells was further evident from increased ratio of cleaved caspase 3 to GAPDH. Thus, knockdown of Cypher had a promote ability on the mitochondria-dependent pathway activity to induce apoptosis in cardiomyocytes.

### MAPK and Akt participated in downregulation of Cypher induced apoptosis in H9c2 cardiomyocytes

Mitogen-activated protein kinases (MAPK) signal transduction pathways are involved in the regulation of apoptosis in cardiac myocytes 18. Thus, we investigated whether these pathways were modified in Cypher knockdown H9c2 cells and western blotting was used to detect the phosphorylation levels of c-Jun NH2-terminal kinase (JNK), extracellular regulated protein kinases 1/2 (Erk1/2) and p38 MAPK, which are key mediators of cardiac apoptosis. As shown in Fig.4G-J, a significantly increased phosphorylated levels of p38 MAPK and decreased phosphorylation of Erk1/2 in Cypher siRNA treated H9c2 cells were detected by western blotting compared with those in the control, whereas there was no increase in the activity of JNK kinases (data not shown). And there was no significant change in the total expression levels of JNK, p38 MAPK and Erk after Cypher knockdown. We next examined whether the survival protein p-Akt was involved in apoptosis as determined by immunoblotting for phosphorylation of either Thr308 or Ser473. As shown in Fig.4K and 4L, Cypher siRNA treatment decreased phosphorylated Akt Ser473 expression, indicating that apoptotic process in Cypher siRNA treated H9c2 cells involved Akt activation. Collectively, these results suggested that knockdown of Cypher aggravated cardiomyocytes apoptosis possibly by regulation Akt/p38 MAPK signaling pathway.

### SC79 reversed the apoptotic detrimental effects induced Cypher-deficiency

**Figure 5:**
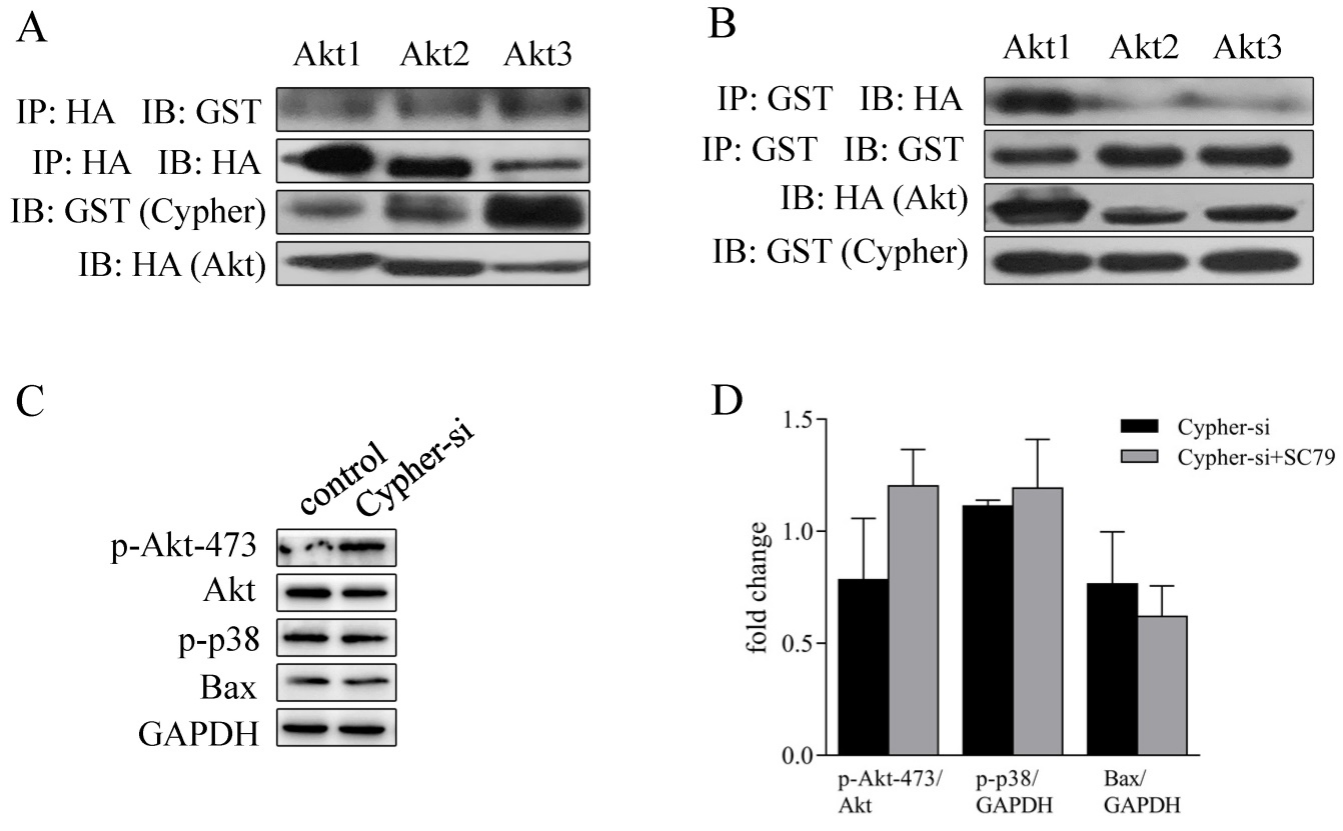
** SC79 inhibited Cypher-siRNA-induced apoptosis of H9c2 cells by activating Akt.** (**A**) HEK293T cells were transfected with GST-Cypher and HA-Akt plasmids. The cells were then lysed. Lysates from HEK293T co-immunoprecipitated (IP) with IgG or antibody to HA, followed by immunoblot (IB) analyses with GST antibody. (**B**) HEK293T cells were transfected with GST-Cypher and HA-Akt plasmids. The cells were then lysed. Lysates from HEK293T IP with IgG or antibody to GST, followed by IB analyses with HA antibody. H9c2 cells were pre-cultured in serum-free medium in the presence of Cypher-siRNA for 72 hrs, and then stimulated further with 10 µM SC79 for an additional 30 mins. The cells were then lysed, and Western blot analysis was performed. (**C**) Western band images are representative of three independent experiments. (**D**) The quantitative analysis of p-Akt-473 using total Akt as normalization, while p-p38 and Bax using GADPH as normalization. Experiments were repeated three times.

Interestingly, coimmunoprecipitation studies with Akt antibodies and Cypher demonstrated their colocalization (**Fig. 5A and 5B**). Then, we focused our attention on the rescue function of Akt activator on knockdown Cypher to reduce cells apoptosis. Finally, H9c2 cells in the presence of Cypher siRNA were then pre-incubated with 10 μM SC79 (a popular used agonist of Akt) for 30 min prior to harvest for western blotting analysis. The effect of the activator was confirmed by significantly increased Akt phosphorylation. Moreover, the increased expressions of apoptotic proteins Bax and p-p38 MAPK caused by Cypher suppression were markedly reversed by Akt activation concurrently. Taken together, these results confirmed that Akt/p38 MAPK constitute an axis regulating apoptosis in the Cypher-deficiency induced cardiomyocyte injury.

## Discussion

DCM, the most primary myocardial disorder, is a major cause of chronic heart failure 19. Various etiologies cause DCM, while the gene mutations paly significant effect on the pathogenesis of DCM 20. Mutations in the sarcomeric and cytoskeletal genes, such as titin, desmin, lamin A/C, and α-actin, accounted for the majority of genetic causes 7. And defects in cytoskeleton damaged cardiac structural integrity which seemed to be a prevalent mechanism of DCM.

Accumulating data indicated the involvement of Cypher/ZASP, one of the cytoskeletal proteins, in the pathogenesis of DCM 8-13. Cypher knockout mice induced a severe form of DCM by disorganizing both sarcomere and cytoskeleton 10. And ablation of c-Jun exhibited similar alterations, such as sarcomere disarrangement, cytoskeleton disorganization, resulting in DCM 21. DCM-associated BAG3 mutations impaired Z-disc assembly and increased the stress-induced apoptosis in cultured cardiomyocytes 22,23. And cardiac tissue from a DCM patient harboring a nonsynonymous mutation in α-actin gene also displayed sarcomeric disarray, and an increase in cardiomyocytes apoptosis 24.

Similarly, Cypher/ZASP not only maintains cardiac structure as scaffold of the cell, but also plays an important role in signal transduction 3. Cypher/ZASP interacts with signaling molecules like PKA, p53, PKCs, and calcineurin to form multiprotein complexes at sarcomeric Z-lines 13-15. However, the specific mechanisms remain unexplored. Since adult myocytes are undifferentiated cells, cardiomyocyte apoptosis has become a major mechanism underlying various cardiovascular diseases, including ischemia/reperfusion injury (I/R), doxorubicin (Dox) induced cardiotoxicity, ischemic heart disease, cardiomyopathy, and ultimately heart failure 25,26. The proposed role for apoptosis in DCM has been supported by accumulating evidence in both human and animal models. Apoptosis rate in DCM patients was 0.08% to 0.25%, much higher than that of the control group (0.001% to 0.002%) 27. And previous studies in both cTnT R141W and LMNA E82K transgenic mice, mouse models of DCM, showed increased apoptosis in the heart 28-30. C-Jun knockout mice also underwent myocardial apoptosis 21. Desmin-deficient mice displayed myocyte apoptosis finally resulting in DCM 31. DCM mutations stress myocytes severely and trigger myocardial apoptosis reducing heart function. We therefore hypothesized that the apoptotic pathway may contribute to the development of DCM induced by Cypher deficient.

**Figure 6:**
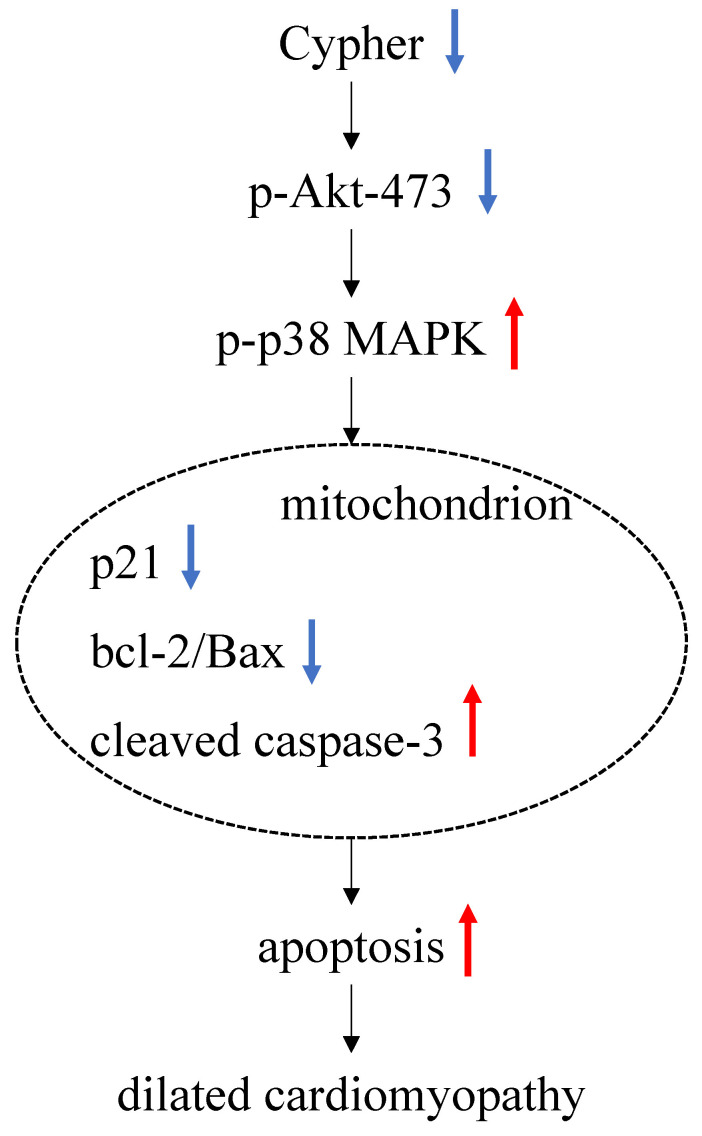
** Schematic of the working signal pathway.** In Cypher deficient cardiomyocytes, phosphor Akt-473 is downregulated and leads to a significant increased p-p38 MAPK. There it is assumed to activate mitochondria pathway, manifested specifically by decreasing the expression of p21 and the ratio of bcl-2 to Bax, while increasing the expression of cleaved caspase-3. These lead to an increase of apoptosis, thus ultimately leading to dilated cardiomyopathy.

Apoptosis, or programmed cell death, is systematically regulated by numerous genes involving the pro- and anti-apoptotic families 32. The mitochondrial pathway, the major apoptosis-inducing pathway, is initiated by releasing cytochrome c (Cyt c) into the cellular cytoplasm to form the apoptosome resulting in activating downstream effector caspase-3 33. The balance between pro-apoptotic (Bax) and anti-apoptotic (bcl-2) protein of the Bcl-2 family, both residing in the outer mitochondrial membrane, plays a crucial role on the process of apoptosis by regulating Cyt c release 34. We then first studied whether the Cypher deficiency in cardiomyocytes exerted a direct apoptotic effect, by performing TUNEL staining, flow cytometric test and measuring caspase-3 activity. As illustrated in **Figure 6**, we observed that knockdown of Cypher significantly decreased cell viability, reduced p21 expression, increased cardiomyocytes apoptosis, the bcl-2/Bax ratio and caspase-3 expression. Furthermore, TUNEL staining in Cypher knockout mice myocardium showed the similar apoptotic effect *in vivo*.

Many stimuli regulate cell apoptosis through the Akt pathway 35. Serine/threonine protein kinase B (PKB)/Akt is involved in the regulation of many process including cell survival, proliferation, metabolism, and angiogenesis 36. Especially, Akt serves as a key mediator in cardiomyocyte apoptosis 37. For example, activation of the Akt has been shown to protect cardiomyocytes from I/R or post-myocardial infarction injury through inhibiting myocyte apoptosis 38,39. Previous studies revealed that Akt was inhibited in Dox-treated hearts, whereas enhanced Akt activation prevented cardiac apoptosis and dysfunction in response to Dox 40. Studies in animal models of DCM revealed the Akt acted as an antiapoptotic effector in heart. Resveratrol improves cardiac function in the ischemic heart diseases and DCM through activating Akt-dependent pathway 41. Dhcr24 protect against DCM also through up-regulation of activated Akt 42. We then investigated whether Cypher-deficiency induced cardiac apoptosis was associated with changes in the Akt survival pathway. In the present study, we found a reduction in phosphorylation in Ser473 of Akt in Cypher knockdown H9c2 cells the same as a notably reduced level of p-Akt was observed in the cTnT R141W mice 29. Previous study showed that phosphorylation Akt at Ser473 was necessary for its full activation 43.

To explore the underlying mechanism, the level of the cell stress signaling Erk, p38 MAPK and JNK were evaluated. Our data showed a significantly decreased level of p-Erk but increased level of p-p38 MAPK in Cypher knockdown H9c2 cells compared to the control group. Finally, to confirm whether the apoptotic effect in H9c2 cells inducing by Cypher deficiency was related to Akt pathway, the Akt activator SC79 was used. We found that SC79 administration protected against apoptosis in H9c2 cells treated with Cypher siRNA by enhancing Akt phosphorylation, inhibiting p38 MAPK phosphorylation and reducing pro-apoptotic protein Bax expression. Thus, there was clear evidence that Akt pathway plays an essential role in apoptotic effects inducing by Cypher deficiency.

Akt and MAPK are two main signaling pathways which participate in cardiomyocyte apoptosis 18,44. The mammalian MAPK family, including Erk1/2, p38 MAPK, and JNK have been identified so far. Increased p38 MAPK activity promotes cardiac apoptosis, which could be suppressed by Akt activation 18. Specifically, p38 MAPK signaling pathway is activated in H2O2-, I/R-, Dox-induced myocardial apoptosis 45-47. Enhanced p38 MAPK activity results in cardiac remodeling and ventricular dilation, leading to severe cardiomyopathy at last. In the contrary to the pro-apoptotic effect of p38 MAPK, Erk1/2 signaling elicits protection in cells to maintain cardiac function by blocking caspase-3 activation 48.

In conclusion, the results of present study suggest that Cypher deficiency induces apoptosis by inhibiting Akt signaling and increasing p38 MAPK phosphorylation, which ultimately leads to DCM.
